# Identifying distinctive tissue and fecal microbial signatures and the tumor-promoting effects of deoxycholic acid on breast cancer

**DOI:** 10.3389/fcimb.2022.1029905

**Published:** 2022-12-13

**Authors:** Na Wang, Jun Yang, Wenjie Han, Mengzhen Han, Xiaolin Liu, Lei Jiang, Hui Cao, Mingxi Jing, Tao Sun, Junnan Xu

**Affiliations:** ^1^ Department of Breast Medicine, Cancer Hospital of China Medical University, Liaoning Cancer Hospital, Shenyang, China; ^2^ Department of Pharmacology, Cancer Hospital of China Medical University, Liaoning Cancer Hospital, Shenyang, China; ^3^ Department of Medicine, Liaoning Kanghui Biotechnology Co., Ltd., Shenyang, China; ^4^ Department of Breast Medicine, Cancer Hospital of Dalian University of Technology, Liaoning Cancer Hospital, Shenyang, China

**Keywords:** breast cancer, 16S rRNA, microbiome, intestine, cancer diagnosis, random forest, *Clostridium*, deoxycholic acid

## Abstract

**Introduction:**

A growing body of evidence indicates that the dysbiosis of both mammary and intestinal microbiota is associated with the initiation and progression of breast tumors. However, the microbial characteristics of patients with breast tumors vary widely across studies, and replicable biomarkers for early-stage breast tumor diagnosis remain elusive.

**Methods:**

We demonstrate a machine learning-based method for the analysis of breast tissue and gut microbial differences among patients with benign breast disease, patients with breast cancer (BC), and healthy individuals using 16S rRNA sequence data retrieved from eight studies. QIIME 2.0 and R software (version 3.6.1) were used for consistent processing. A naive Bayes classifier was trained on the RDP v16 reference database to assign taxonomy using the Vsearch software.

**Results:**

After re-analyzing with a total of 768 breast tissue samples and 1,311 fecal samples, we confirmed that *Halomonas* and *Shewanella* were the most representative genera of BC tissue. *Bacteroides* are frequently and significantly enriched in the intestines of patients with breast tumor. The areas under the curve (AUCs) of random forest models were 74.27% and 68.08% for breast carcinoma tissues and stool samples, respectively. The model was validated for effectiveness *via* cohort-to-cohort transfer (average AUC =0.65) and leave-one-cohort-out (average AUC = 0.66). The same BC-associated biomarker *Clostridium_XlVa* exists in the tissues and the gut. The results of the *in-vitro* experiments showed that the *Clostridium*-specific-related metabolite deoxycholic acid (DCA) promotes the proliferation of HER2-positive BC cells and stimulates G0/G1 phase cells to enter the S phase, which may be related to the activation of peptide-O-fucosyltransferase activity functions and the neuroactive ligand–receptor interaction pathway.

**Discussion:**

The results of this study will improve our understanding of the microbial profile of breast tumors. Changes in the microbial population may be present in both the tissues and the gut of patients with BC, and specific markers could aid in the early diagnosis of BC. The findings from *in-vitro* experiments confirmed that *Clostridium*-specific metabolite DCA promotes the proliferation of BC cells. We propose the use of stool-based biomarkers in clinical application as a non-invasive and convenient diagnostic method.

## Introduction

Breast cancer (BC) is a common type of cancer and accounts for most cancer-related deaths in women worldwide. According to the latest cancer statistics report, an estimated 2,261,419 new cases (11.7% of total cancer cases) and 684,996 cancer deaths (6.9% of total cancer deaths) were reported in women worldwide in 2020 (Sung et al., 2021). In recent years, the BC incidence rates have continued to increase at an annual rate of 0.5% per year (Siegel et al., 2021). The findings of a hallmark population-based study on cancer indicated that early-stage detection can help reduce breast cancer mortality by one-third (Elmore et al., 2005; Jafari et al., 2018). As the availability of high-throughput sequencing technology has continued to expand and its cost has decreased, the identification and characterization of the tissue or gut microbiome in patients with BC have constituted an active area of research. In addition to the various imaging techniques and biochemical parameters, tumor-related microbes may be used as diagnostic biomarkers for breast diseases, and investigating the microbiome could be a novel cancer diagnostic method.

Our perception of sterility in breast tissue has undergone significant changes over time. The breast microbiome may originate from the intestine and is highly malleable (Shively et al., 2018; Chadha et al., 2021). Recent advances in metagenomics and next-generation sequencing technologies have promoted a more comprehensive understanding of the complex relationships between human microbiota and breast disease. We confirmed that, across different studies, mammary gland tissues were found to harbor disparate microbial communities, some even exhibiting contrasting microbial abundances. Hieken et al. confirmed that *Fusobacterium*, *Gluconacetobacter*, *Lactobacillus*, and *Atopobium* were significantly enriched in breast cancerous tissues compared with those in healthy individuals (Hieken et al., 2016). Malignant tumor tissues also showed an increase in the relative abundance of *Ralstonia*, and a relative decrease in the abundance of *Methylobacterium* was also observed compared with that in healthy controls in another study (Costantini et al., 2018). However, Xuan et al. showed *Methylobacterium* to have the greatest prevalence in malignant tumor tissues (Xuan et al., 2014). The gut microbiome, as one of the major sources of the breast microbiome, contributes to physiological immune modulation and serves as a critical component in the occurrence and progression of mammary carcinoma (Zhang et al., 2020; Zhang et al., 2021). Similarly, previous studies have not reported the same findings on intestinal microorganisms in patients with breast diseases. Studies focused on population-based community metrics have shown that the populations of *Coprococcus*, *Butyricimonas*, and *Odoribacter* in fecal samples were most inversely associated with BC (Bobin-Dubigeon et al., 2021). In other studies, there is a high abundance of *Bacteroides*, *Clostridiaceae*, *Ruminococcaceae*, and *Faecalibacterium* in stool samples collected from patients with BC, along with a low abundance of *Romboutsia*, *Coprococcus*, *Dorea*, and *Lachnospiraceae* (Goedert et al., 2015; Byrd et al., 2021). Substantial variability in the abundances of microbial communities has been reported in different studies, possibly owing to various biological factors, such as individual genetic variance, ethnicity, and dietary habits that influence microbiota composition. Furthermore, the presence of hypervariable regions, the sequencing platform and the bioinformatics pipelines used, and the method of data processing differ from study to study, thus making tissue and fecal microbiome more challenging to analyze uniformly and systematically.

Meta-analysis serves as a useful research tool owing to its informative and unbiased nature, which helps reduce the effect of biological and technological differences across multiple studies and provide stabilized and accurate results (Gurevitch et al., 2018). Several meta-analyses have been performed for the identification of microbial markers in colorectal cancer based on 16S rRNA or shotgun genome sequencing datasets (Sze and Schloss, 2018; Mo et al., 2020; Wu et al., 2021). An urgent need exists to explore and precisely identify stool- and tissue-based microbial biomarkers. While findings from certain studies have confirmed that the microbial communities differ significantly between patients with breast diseases and healthy controls, the co-differences of microbes between these two body sites remain unclear.

In this study, we performed an integrated analysis using public 16S rRNA sequence data from both breast tissues (*n* = 768) and stool samples (*n* = 1,311) from healthy women and women with breast diseases across eight studies to improve our understanding of specific microbial community in breast diseases and specific microbe-related metabolics in breast carcinogenesis. *Clostridium_XlVa* had simultaneously high expression both in breast tissue and gut environment. DCA was the main and specific metabolic biotransformed by *Clostridium* with primary bile acids and reabsorbed in the colon and recycled to the liver, then conjugating to glycine or taurine, similar to primary bile acids inducing biliary lipid secretion, and solubilizing cholesterol in the bile, contributing approximately 20% to the bile acid pool (Chiang, 2013). Yoshimoto et al. revealed that the levels of DCA and *Clostridium* genus increased simultaneously in high-fat diet mice by liquid chromatography–mass spectrometry (LC-MS) and 16S rRNA analysis (Yoshimoto et al., 2013). Considering the specific 7α-dehydroxylating activity carried by the *Clostridium* genus, the explanation for it is that the *Clostridium* genus contributes to an increase in the DCA level. DCA could promote M1 macrophage polarization and pro-inflammatory cytokine production (Wang et al., 2020) and be involved in gastrointestinal cancer dose-dependently (Nagathihalli et al., 2014). DCA exposure could inhibit farnesoid X receptor expression and enhance the Wnt–β-catenin signaling pathway, thereby contributing to colon carcinogenesis (Yao et al., 2022). In addition, the enterohepatic circulation of DCA provokes senescence-associated secretory phenotype in hepatic stellate cells, facilitating hepatocellular carcinoma development (Yoshimoto et al., 2013). These notions prompted us to examine if *Clostridium-*specific-related DCA has key roles in BC development. Therefore, DCA was used to demonstrate the effect of specific microbe-related metabolics on BC cell growth and apoptosis. Based on these results, we combined the method with proteomic analysis to investigate the mechanisms by which microbial metabolites contribute to BC development. In addition, after pooling data from different cohorts, we constructed a random forest (RF) model to distinguish patients with BC from non-cancer controls. Cohort-to-cohort transfer validation and leave-one-cohort-out (LOCO) validation across multiple datasets were employed to overcome the influence of technical discrepancies and cohort heterogeneity. Collectively, our study suggests that distinct microbiota populations are present in breast tissues or gut of patients with BC and non-cancer controls, the RF model constructed based on specific members of the microbial community can more accurately classify patients with BC, and *Clostridium*-specific DCA could stimulate BC cell growth.

## Materials and methods

### Study selection

PubMed was searched for studies (published on or before up to 01 January 2022) that used breast tissue or stool microbiota obtained from patients with breast disease using the terms “(Breast Neoplasms or breast cancer) AND (microbiome or microbiota) AND (metagenomic or mNGS or 16S rRNA).” The search was limited to studies that fit the following criteria: 1) had original data, 2) used fecal or breast tissue samples, and 3) included breast disease and normal control populations. Systematic reviews, letters to the editors, conference papers, case reports, and meta-analyses data were excluded. Of the nine studies that fulfilled the criteria, in eight studies, the 16S rRNA gene was characterized, and in only one study, shotgun metagenomics was used. The eight studies that characterized the 16S rRNA gene were included in this meta-analysis.

### Data retrieval and processing

Raw sequencing data were retrieved from the National Center for Biotechnology Information Sequence Read Archive (NCBI-SRA) database using the following accession numbers (BioProject ID): PRJNA624822 for Nejman et al. (2020), SRP076038 for Urbaniak et al. (2016), PRJNA335375 for Hieken et al. (2016), PRJNA637875 for Thyagarajan et al. (2020), PRJNA383849 for Goedert et al. (2018), PRJNA658160 for Byrd et al. (2021), PRJNA566060 for Yao et al. (2021), and PRJNA630839 for Guan et al. (2020). The downloaded FASTQ data of each study were reprocessed separately with consistent processing to avoid the biases arising from bioinformatics analyses. All 16S rRNA gene datasets containing forward and reverse read files were processed using a standardized pipeline in QIIME 2.0 after an initial visualization of read quality using the FastQC and MultiQC software. The DADA2 (V.2018.11) software, wrapped in QIIME2, was used to filter sequencing reads with quality score *Q >*25 and denoise reads into amplicon sequence variants, which resulted in the formation of feature tables and representative sequences for each study.

### Combined processing

Feature tables and representative sequences in each study were merged using QIIME2’s merge and merge-seqs commands. R software (version 3.6.1) was used to conduct further analyses using the data exported from QIIME2. A naive Bayes classifier was trained on the RDP v16 reference database to assign taxonomy using the Vsearch software. Sequences that were identified as “Chloroplast,” “Mitochondria,” and those that were unclassified at the kingdom level were removed from the datasets. Samples that could not be mapped to any species were omitted. ANOVA-like analysis was used to quantify the effect of potential confounding factors and disease status. The total variance within the abundance of a given ASV was compared to the variance explained by disease status (BC, benign tumor, and normal) and the variance explained by confounding factors including study, age, and BMI. Variance calculations were performed on ranks in order to account for the non-Gaussian distribution of microbiome abundance data. Potential confounders were transformed into categorical data (study) as quartiles (age) and for the case of BMI into lean (>25), obese (25–30), and overweight (>30). Beta diversity in microbial composition was calculated using the Bray–Curtis dissimilarity metrics, and principal coordinate analysis (PCoA) was performed based on the Bray–Curtis dissimilarity matrix using the R “vegan” package. Microbiome data were CLR-transformed using the “compositions” package in R software. The significance of differential abundance was tested on genera using the STAMP software after CLR transformation of microbiome data (Welch’s *t*-test and *P*-values <0.05 were considered significant). BC-related biomarkers were identified by the Wilcoxon rank-sum tests based on CLR-transformed data. Genera with *P <*0.05 and high abundance in the BC group were considered as BC-related biomarkers.

### RF model construction and evaluation

Based on the sequence information, we prepared RF models using the “randomForest” package with default parameters to distinguish the cancer status from the non-cancer status. Stratified 10-fold cross-validation was used to configure the training and testing datasets. The minimum error was calculated using five-fold cross-validation with the “rfcv” function. Based on the Mean-Decrease-Accuracy values, the model was constructed using the top 30 most important genera, which are considered as important microbial characteristics. LOCO and cohort-to-cohort validation were performed to evaluate the generalizability of microbe-based BC classifiers, including geographic variation and technical differences in the 16S rRNA microbial data across multiple studies. In LOCO validation, data from one cohort were used as the testing set to assess the training set, which was set by pooled data from the remaining cohorts. In the cohort-to-cohort validation, we constructed the RF classifier models using a single cohort and used other cohorts as the testing data separately to evaluate classifiers. In addition, a nested cross-validation was used on the training study to calculate the within-study accuracy.

### Cell line culture and the configuration of metabolite solution

The human breast cancer cell lines MDA-MB-231 and SK-BR-3 were purchased from the ATCC (ATCC, Rockville, MD, USA) and cultured in complete DMEM (12800-058; Thermo Fisher Scientific, Inc., China) supplemented with 10% fetal bovine serum (FBS; C04001-500; Biological Industries, China) and 1% penicillin/streptomycin (P1400, Solarbio, China) at 37°C in 5% CO_2_. DCA (D103698; Aladdin, China) was dissolved in DMSO (DH105-2; Dingguo Biology, China), and a stock solution of 250 mM was stored at −20°C.

### Cell proliferation assay

Growth assays were performed using a Cell Counting Kit-8 (CCK8) assay kit (CK04; Dojindo Laboratories, Japan). Briefly, MDA-MB-231 and SK-BR-3 cells were seeded in 96-well plates at 10^3^ cells/well and cultured for 24 h and then treated with DCA for 24 h. After treatment, CCK8 was added to each well (10 μl of CCK8 substrate and 90 μl of complete DMEM), and the cells were incubated at 37°C for 1 h. The optical density (OD) was determined using a microplate reader at 450 nm (Multiskan MK3; Pioneer Co-operative UK Ltd., Britain). After normalization, cell viability was calculated using the formula: Cell viability = (OD value of the experimental well − OD value of the blank well)/(OD value of the control well − OD value of the blank well) × 100%.

### Apoptosis analysis

We detected apoptosis by Annexin V FITC/PI staining. SK-BR-3 cells were seeded in six-well plates (105 cells/well), cultured for 24 h, and then treated with DCA (0, 40, 60, and 80 μmol/L) for 24 h. Following this, SK-BR-3 cells were harvested and resuspended in 500 μl of binding buffer. Five microliters of Annexin V/FITC (WLA001c; Wanleibio, China) and 10 μl of PI solution were added and incubated in the dark for 15 min at 25°C. The cells were assessed using a flow cytometer (BD FACSAria II; BD Co., USA), and data were analyzed using the FlowJo software.

### Cell cycle analysis

Cell cycle analysis was performed using flow cytometry. Briefly, SK-BR-3 cells were seeded in six-well plates (10^5^ cells/well) and incubated for 24 h in DMEM supplemented with DCA (0, 40, 60, and 80 μmol/L). The cells were then harvested, centrifuged (1,500 rpm, 5 min), and fixed overnight with 70% precooled ethanol at 4°C and resuspended in ice-cold phosphate-buffered saline (PBS). Subsequently, 100 μl of RNase A solution (WLA010a; Wanleibio, China) was added to the cell suspension, and the cells were incubated for 30 min at 37°C in a water bath. Five hundred microliters of PI was added to the cells, which were incubated in the dark at 4°C for 30 min. A flow cytometer was used to determine the cell cycle of the cells, and data were analyzed using the FlowJo software.

### Proteomics analysis

SDT buffer (4% SDS, 100 mM of Tris–HCl, pH 7.6) was added to the sample. The lysate was sonicated and boiled for 10 min. After centrifugation at 14,000*g* for 15 min, the protein content in the supernatant was quantified using the BCA Protein Assay Kit (P0012, Beyotime, China). The proteins were separated on a 12% SDS-PAGE gel. One hundred microliters of iodoacetamide (IAA) (100 mM of IAA in UA buffer) was added to block the reduced cysteine residues, and the samples were incubated for 30** min** in the dark. Finally, the protein suspensions were digested using 4 μg of trypsin (V5117, Promega, China) in 50 mM of NH_4_HCO_3_ buffer overnight at 37°C. The peptide segment was desalted using a C18 column. The peptide content was estimated based on the UV light spectral density at 280 nm using an extinction coefficient of 1.1 in a 0.1% (g/L) solution.

### Analysis of mass spectrometry data

The samples were analyzed on a nanoElute (Bruker, Bremen, Germany) coupled to a timsTOF Pro (Bruker, Bremen, Germany) equipped with a CaptiveSpray source. Peptides were separated on a 25-cm × 75-μm analytical column, with 1.6 μm of C18 beads with a packed emitter tip (IonOpticks, Australia). The column was equilibrated using 4 column volumes before loading the sample in 100% buffer A (99.9% Milli-Q water, 0.1% FA). The samples were separated at 300 nl/min using a linear gradient as follows: 1.5 h gradient: 2%–22% buffer B for 75 min, 22%–37% buffer B for 5 min, 37%–80% buffer B for 5 min, and hold in 80% buffer B for 5 min.

### Data analysis

The MaxQuant software was used to analyze the MS data. A maximum of two missed cleavage sites and a mass tolerance of 40 ppm for fragment ions were allowed. The cutoff of the global false discovery rate (FDR) for peptide and protein identification was set to 0.01. Protein abundance was calculated based on the normalized spectral protein intensity (LFQ intensity). Protein fold change >2 or <0.5 and *P*-value (Student’s *t*-test) <0.05 were considered to indicate differentially expressed proteins.

### Bioinformatics analysis

All protein sequences were aligned to those present in the NCBI BLAST+. The GO terms of the sequences were selected using the top Bit-Score by Blast2GO. Following this, the annotation from GO terms to proteins was completed. Fisher’s exact test was used to enrich the GO terms by comparing the number of differentially expressed proteins and total proteins correlated with the GO terms. The KEGG database was used for pathway analysis. Fisher’s exact test was used to identify the significantly enriched pathways.

### Statistical analysis

Each experiment was performed at least three times to ensure reproducibility. Results are presented as mean ± SEM. Significance was calculated using one-way ANOVA followed by Bonferroni’s multiple comparisons test. GraphPad Prism 8.0 was used to perform all statistical analyses. Statistical significance was considered at *P <0.05 and **P <0.01.

## Results

### Characteristics of the datasets in the meta-analysis

Eight studies that employed the 16S rRNA sequencing method for microbiome analysis of tissue and fecal samples from patients with benign and malignant breast disease and healthy controls were selected in this meta-analysis to characterize the distinct microbial characteristics. Among the studies, four were conducted in the USA, two in China, one in Israel, one in Canada, and one in Ghana. Notably, we only included data obtained from breast tissue samples from studies conducted by Nejman et al. and Hieken et al., and data from non-breast and non-tissue samples were excluded. In total, we pooled data from eight studies, which included data from 768 tissue samples and 1,311 fecal samples. The sample sizes and clinicopathological data from the datasets for each cohort are presented in **Table 1**. After quality control using the DADA2 method, data satisfying the follow-up requirements for subsequent analysis were included. A summary of sequences analyzed by DADA2 is listed in **Table 2**.

**Table 1:** Details of the large-scale breast tissue or gut datasets included in this study.

Study	Type	Group (*n*)	Age (average ± SD)	BMI (average ± SD)	Gravidity Yes (%)	Menopausal Yes (%)	Platform	Region	Country
Nejman (2020)	Tissue	Normal (51)	46.3 ± 14.5	–	–	–	Illumina_Hiseq, Illumina_Miseq, Illumina_NextSeq	V2, V3, V5, V6, V8	Israel
		Benign (29)	41.7 ± 11.9	–	–	–			
		Cancer (35)	57.6 ± 12.6	–	–	–			
		Cancer-adjacent (173)	57.6 ± 12.1	–	–	–			
Urbaniak (2016)	Tissue	Normal (23)	48.9 ± 12.1	–	17 (73.9%)	12 (52.2%)	Illumina_MiSeq	V6	Canada
		Benign (13)	55.6 ± 16.7	–	6 (46.2%)	2 (15.4%)			
		Cancer (32)	54.7 ± 17.0	–	28 (87.5%)	29 (90.6%)			
Hieken (2016)	Tissue	Benign (12)	60.8 ± 13.2	29.5 ± 7.0	11 (91.7%)	6 (50.0%)	Illumina_MiSeq	V3–V5	USA
		Cancer (16)	62.8 ± 12.1	30.1 ± 6.4	13 (81.3%)	14 (87.5%)			
Thyagarajan (2020)	Tissue	Cancer (33)	25.0–78.0	–	–	–	Illumina_MiSeq	V3–V4	USA
		Cancer-adjacent (31)	25.0–78.0	–	–	–			
Goedert (2018)	Stool	Normal (144)	62.0 ± 5.2	28.2 ± 5.5	–	144 (100.0%)	Illumina_MiSeq	V4	USA
		Cancer (144)	61.0 ± 3.1	28.3 ± 5.8	–	144 (100.0%)			
Byrd (2021)	Stool	Normal (442)	46.9 ± 12.9	28.0 ± 7.9	391 (88.5%)	189 (42.8%)	Illumina_MiSeq	V4	Ghana
		Benign (111)	38.8 ± 12.8	27.7 ± 6.5	77 (69.4%)	28 (25.2%)			
		Cancer (403)	50.8 ± 12.3	27.1 ± 5.9	348 (86.4%)	226 (56.1%)			
Yao (2021)	Stool	Cancer (36)	–	–	–	–	Illumina_HiSeq	V3–V4	China
Guan (2020)	Stool	Cancer (31)	–	–	–	7 (22.6%)	Illumina_MiSeq	V4	China

**Table 2:** The number of reads produced by each step during quality control.

Cohorts	No. of samples	Sequence lengths (bp)	No. of reads				
			Input	Filtered	Denoised	Merged	Non-chimeric
Nejman (2020)	608	150-PE	121,500,721	117,747,260	117,546,602	98,076,145	95,936,476
Urbaniak (2016)	68	100-SE	901,286	899,895	885,093	–	878,656
Hieken (2016)	28	300-SE	4,571,009	3,840,371	3,828,956	–	3,664,393
Thyagarajan (2020)	64	300-PE	1,800,520	1,009,714	977,573	906,868	352,481
Goedert (2018)	288	250-PE	8,580,928	6,294,134	6,204,855	5,616,720	5,527,051
Byrd (2021)	956	150-PE	25,036,506	24,081,617	23,828,549	21,995,066	19,955,146
Yao (2021)	36	250-PE	10,636,634	9,953,346	9,825,646	8,848,662	1,279,577
Guan (2020)	31	150-PE	1,959,200	1,916,553	1,905,425	1,875,691	1,805,792

### Confounder analysis of the microbiome associated with BC

**Figure 1:**
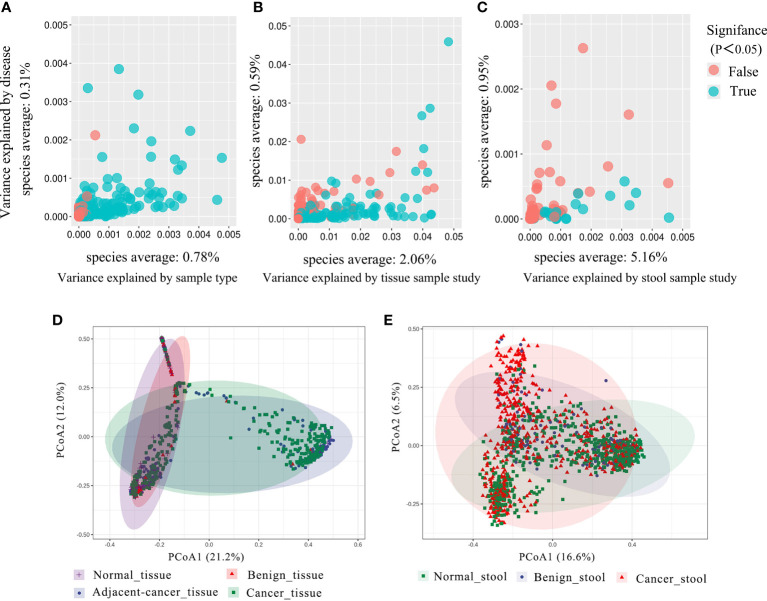
The total variance explained by disease status (BC, benign tumor, health) is plotted against total explained by sample type **(A)**, study of tissue **(B)**, and study of gut **(C)** for individual ASVs. The significantly differential ASVs are colored in blue and *P*-values were from the two-way ANOVA test. **(D, E)** Principal coordinate analysis (PCoA) of the breast tissue and gut microbiome based on the Bray–Curtis metric. Each ellipse in different color represents the 95% confidence level. **(D)** In breast tissue samples, the purple and red ellipses are similar, and the blue and green ellipses are almost overlapping, indicating insignificant differences between normal and benign tissues, adjacent-cancer, and cancer tissue. **(E)** The ellipses that represented normal, benign, and cancer stool microbiota are inconsistent, demonstrating significant differences among different groups.

As studies differed from one another in many biological aspects, we first investigated the effect of potential confounders (including sample type, study, patients’ age, BMI). This analysis revealed that “sample type” has a predominant impact on species composition (**Figure 1A**), and analyzing the microbiome in different sample types separately is essential. Additionally, we quantified the effect of age, BMI, and study on microbiome composition and contrasted this with disease status (**Figures S1, S2**). The variance explained by “study” was greater than that by disease status and by other potential confounders. Study heterogeneity has a large effect on overall microbiome composition (**Figures 1B, C**).

### Alterations in microbial composition in BC

Analysis based on Bray–Curtis dissimilarity indicated the extensive variations in the different sample groups. For the tissue microbiota, PCoA showed distinct distributions among normal breast tissue, benign breast tissue, breast carcinoma tissue, and lesion-adjacent cancer tissue (**Figure 1D**). Furthermore, this finding also indicated a clearer distinction between tissues from cancer (including cancerous and adjacent tissues) and non-cancer (including benign diseases and normal breast tissue) groups. For the stool microbiota, the ecological discrepancy among the gut microbiota from patients with breast cancer and benign breast tumor and the healthy population was distinguishable (**Figure 1E**).

**Figure 2:**
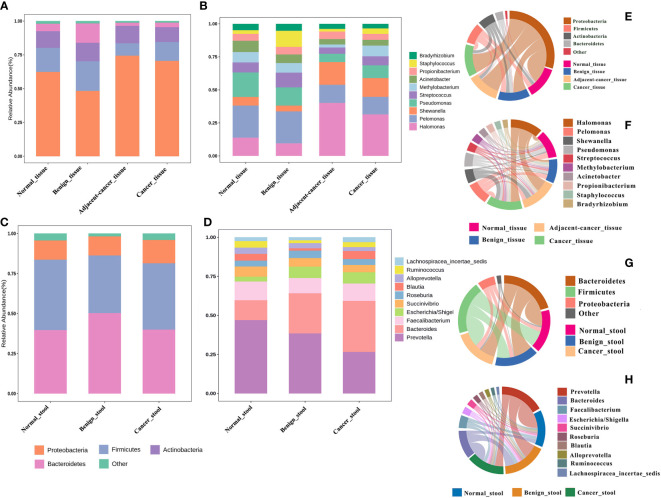
Relative proportions of bacteria in the breast tissue and gut environment of healthy controls, benign breast tumors, and breast cancer. **(A, B)** The bacterial abundance at the phylum and genus levels in breast tissues, respectively, and the structural analysis of the intestinal microbiome are displayed in **(C)** and **(D)**. (**E–H**) Chord plots of the relative abundance at the phylum and genus levels in the tissues and gut.

The idea of defining a core microbiota in breast tumor tissues or gut of patients with BC is intriguing because of its potential association with carcinogenesis. In the current meta-analysis, the four most abundant phyla in all groups were Proteobacteria, Firmicutes, Actinobacteria, and Bacteroidetes, with relative abundances depending on the tissue type. Compared with that in normal and benign tissues, the Proteobacteria phylum was slightly more abundant in cancer tissues and lesion-adjacent tissues, accounting for 70.5% and 74.4% of all bacteria. Meanwhile, the abundance of Bacteroidetes was lower (3.4% in the cancer group and 2.3% in the lesion-adjacent tissue group) (**Figure 2A**). Relative to normal and benign tissues, cancer and lesion-adjacent tissues exhibited a greater proportion of *Halomonas* and *Shewanella* and a lesser proportion of *Pelomonas* and *Pseudomonas* at the genus level (**Figure 2B**). Gut bacterial communities were distinct from the tissue microbial communities, and Bacteroidetes, Firmicutes, and Proteobacteria were the major bacterial phyla (**Figure 2C**). In the gut samples obtained from patients with cancer, the abundance of bacteria from the *Prevotella* genus was found to decline, whereas the relative abundance of the *Bacteroides* genus increased, accounting for 19.5% of the total genera identified (**Figure 2D**). The microbiota from different types of breast tumors have distinct compositions in the tissue or intestinal environment (**Figures 2E, H**).

### Differential abundance analysis identifies significantly enriched and depleted features

**Figure 3:**
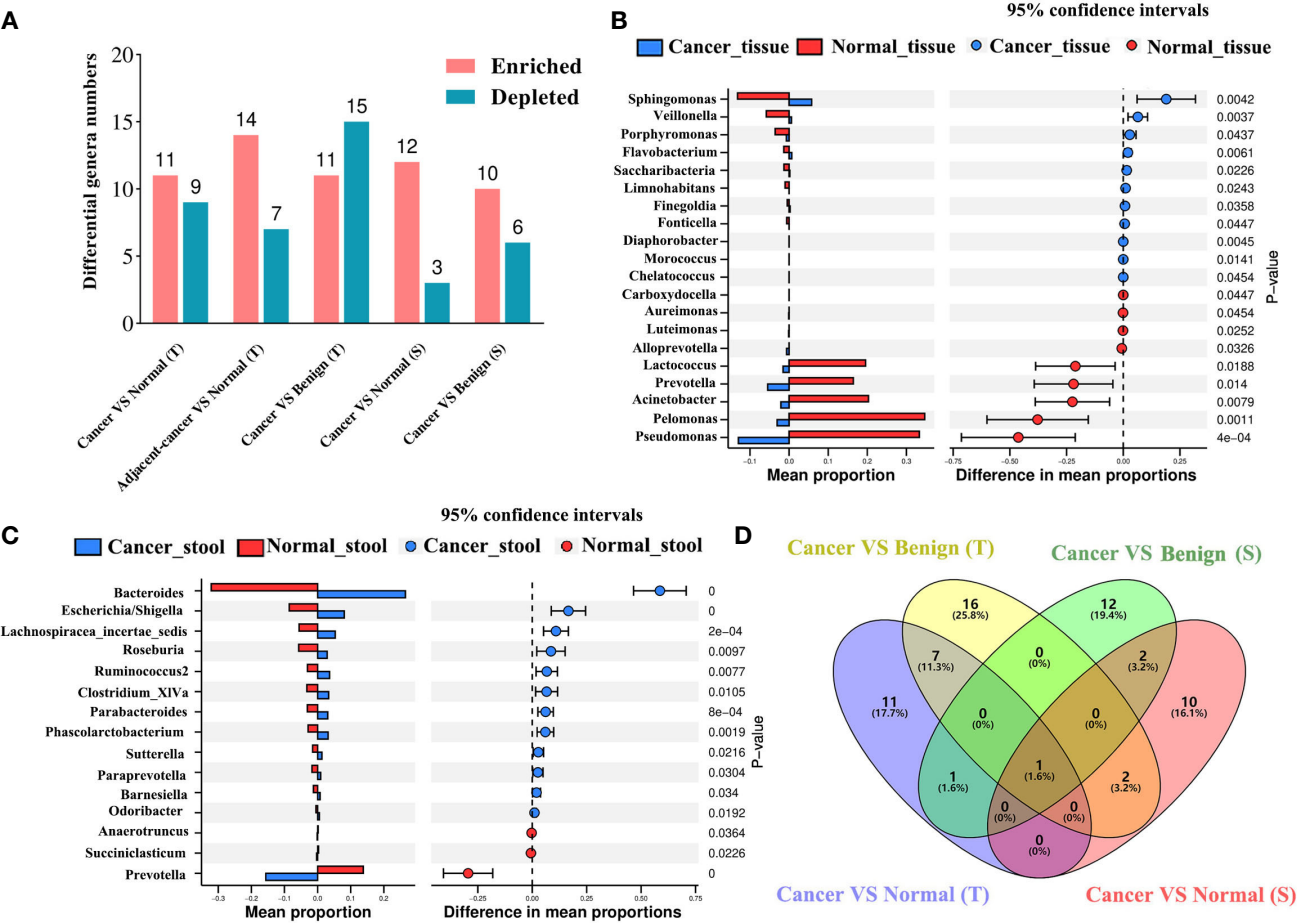
Differential microbial analyses show the enriched and depleted genera identified using different strategies. **(A)** The differential microbial numbers in each comparison strategy. Differential abundance analysis was conducted using the Welch’s *t*-test based on CLR-transformed data and *P*-value <0.05 considered enriched or depleted. VS: comparison of the former to the latter; enriched or depleted genera were identified based on the latter. Difference in the abundance of tissue **(B)** and gut **(C)** bacteria between patients with BC and healthy controls. The blue color presents the abundance of bacteria in the BC group, and the red color presents the abundance of bacteria in healthy controls. Venn plot **(D)** displays the overlap of the genera among BC vs. normal or benign disease strategies in breast tissue and stool samples. BC, breast cancer; T, tissue; S, stool.

We deployed five differential abundance analysis groups, namely, cancer tissue vs. normal tissue, lesion-adjacent tissue vs. normal tissue, cancer tissue vs. benign tissue, cancer stool vs. normal stool, and cancer stool vs. benign stool, to investigate the potential differences in the microbiota. Compared with those in normal tissues, *Sphingomonas*, *Veillonella*, *Porphyromonas*, and *Flavobacterium* were found to be significantly enriched in cancer tissues, whereas nine genera were found to be significantly depleted (**Figures 3A, B**). In the lesion-adjacent tissue vs. normal tissue group, 14 enriched and 7 depleted genera were identified in lesion-adjacent tissues (**Figure S3**). Eleven enriched and 15 depleted genera were identified in the cancer tissues compared with those in the benign tissues (**Figure S4**). Twelve enriched genera (including *Bacteroides*, *Escherichia*/*Shigella*, *Lachnospiracea_incertae_sedis*, *Roseburia*, *Ruminococcus2*, *Clostridium_XlVa*, *Parabacteroides*, and *Phascolarctobacterium*) and *Prevotella*, *Succiniclasticum*, and *Anaerotruncus* were observed in the intestinal microbiome, analyzed using stool samples from patients with cancer compared with those of healthy controls (**Figure 3C**). Compared with those in the stool samples of patients with benign disease, 10 enriched genera and 6 depleted genera were identified in the stool samples of patients with cancer (**Figure S5**). This led us to identify *Prevotella*, which was depleted in patients with BC compared with that in the non-cancer group and constituted an overlapping differential characteristic in both tissue and stool sampling (**Figure 3D**).

### Microbial classification models for BC and non-cancer controls

**Figure 4:**
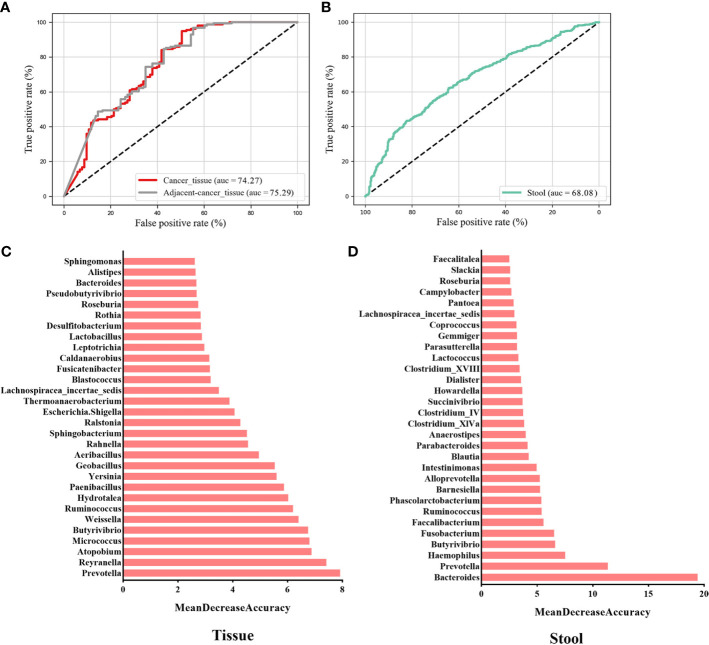
Key components of the random forest (RF) model constructed to distinguish BC from non-cancer controls. The AUC of the BC vs. non-cancer RF model constructed with key genera in breast tissues **(A)** and the gut **(C)** environment. The rank in **(B)** and **(D)** indicates the order of feature importance in the tissue and gut microbial RF model. BC, breast cancer.

To evaluate whether the microbial information can be used to distinguish cancer from non-cancer controls, we established 10-fold cross-validation RF classifiers by pooling tissue and stool samples, respectively. Here, benign and normal samples were classified into one category named “non-cancer.” The RF model (AUC = 74.27%) was constructed using the top 30 most important genera in the tissue samples (**Figure 4A**). The most important key microbial features were *Prevotella*, *Reyranella*, *Atopobium*, *Micrococcus*, *Butyrivibrio*, *Weissella*, *Ruminococcus*, *Hydrotalea*, *Paenibacillus*, and *Yersinia*, among others (**Figure 4B**). To determine whether cancer-adjacent tissues also play a pivotal role in distinguishing BC, we reconstructed the RF model using cancer-adjacent tissue and a non-cancerous microbiome, using the top 30 important genera in the cancerous vs. non-cancerous tissue model as the input for our new model. The AUC was 75.29% (**Figure 4A**). We noticed that the classifiers performed similarly in cancer vs. control and cancer-adjacent vs. control groups, likely because the cancer-adjacent tissue microbiome closely resembles that of cancerous tissues. This indicated that adjacent tissues also possess the ability to diagnose BC. Additionally, when stool microbial characteristics were used to build the model for predicting cancer, the AUC was 68.08%, and the top 30 important features belong to *Bacteroides*, *Prevotella*, *Haemophilus*, *Butyrivibrio*, *Fusobacterium*, and *Faecalibacterium* (**Figures 4C, D**; **Tables S1, S2**).

### Cohort-to-cohort and LOCO validation of BC classifiers

**Figure 5:**
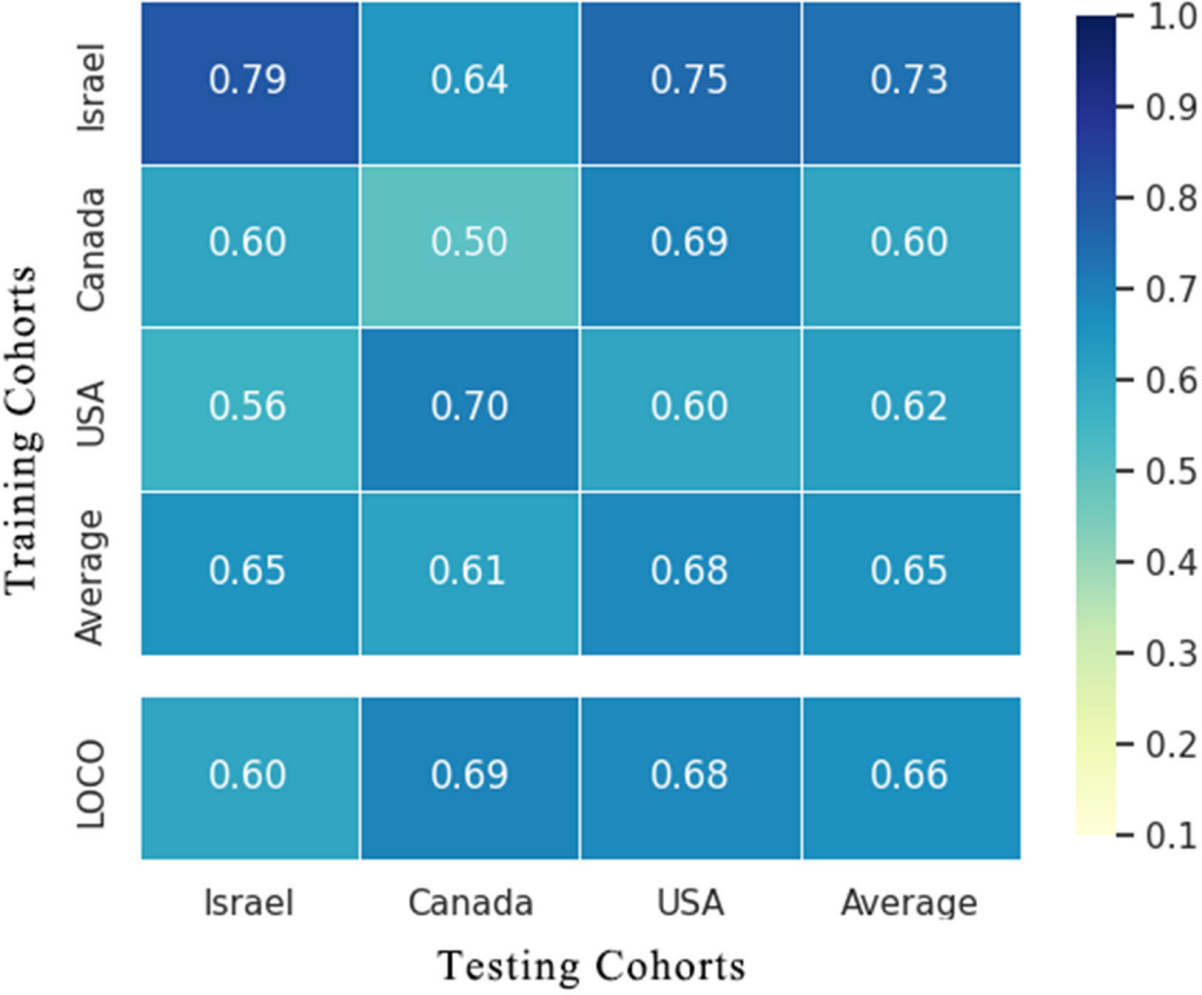
Cross-prediction matrix detailing the prediction AUC values for the four cohorts themselves and between them for the prediction of BC using important features. Values on the diagonal refer to the results of cross-cohort validation within each study (70% of the samples were randomly selected as the training set, whereas the remaining 30% were used as the testing set). The non-diagonal AUC values were obtained by training the classifier on the study in each row and tested on the study in the corresponding column. In the LOCO validation, data from one cohort (column) were used as the testing set to assess the training set, which were set by pooled data from the remaining cohorts. BC, breast cancer.

To evaluate whether the identified essential characteristics were reproducible across multiple studies, we conducted LOCO and cohort-to-cohort transfer validation by pooling data from cancerous and non-cancerous tissue samples. Cohort-to-cohort transfer validation was performed using the cancer stool vs. non-cancer stool strategy because only two cohorts were available. In the cancer tissue vs. non-cancer tissue RF models, the AUCs of cohort-to-cohort transfer validation ranged from 0.50 to 0.79, with an average of 0.65, whereas those in the LOCO analysis ranged from 0.60 to 0.69 (average AUC = 0.66) (**Figure 5**). Notably, the dataset provided by Nejman et al. was a better training set than those obtained from other studies and achieved a relatively higher testing AUC (average AUC = 0.73). When leaving the Nejman_Israel cohort out as the independent validation, the AUC decreased significantly compared with that of the other groups. This may be explained by the larger size of the dataset. Prediction models constructed using a larger sample size could help identify the signature signals of microorganisms associated with BC.

Models prepared using stool microbes could also be used to distinguish BC from healthy individuals. However, some weakness was noted in the cross-cohort validations. When the cohorts Goedert_USA and Byrd_Ghana were used as the validation datasets, respectively, and another cohort was used as the training dataset, the AUCs of the model were 0.54 and 0.59. This result suggests that low AUC values may be attributed to different geographical locations, and the assessment of characteristic gut microorganism communities requires accounting for geographical heterogeneity in dietary habits across different areas. Our results validated the predictive ability and stability of the RF models prepared using important microbial genera as the feature set for BC diagnosis. Furthermore, geographic region, sample type, and sample size impact the diagnostic ability of the RF models.

### *Clostridium*-specific-related DCA stimulates proliferation by promoting cell cycle entry into the S phase in HER2-positive BC cells

**Figure 6:**
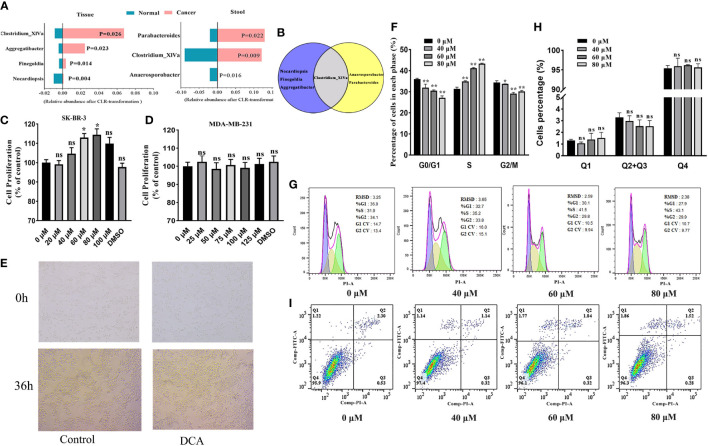
**(A)** The BC-related biomarkers in breast tissue and the gut environment (only BC and healthy controls are considered here). Venn plot **(B)** displays the *Clostridium_XlVa* as the overlap biomarker in both breast tissue and the gut environment. The SK-BR-3 and MDA-MB-231 cells were cultured in DMEM for 12 h and then treated with various concentrations of deoxycholic acid (DCA). The bar graph shows the percentage of viable SK-BR-3 **(C)** and MDA-MB-231 **(D)** cells after treatment with DCA for 24 h, as determined in the CCK8 assay. **(E)** Light microscopy showed proliferation-related changes in SK-BR-3 cells treated with 80 μM of DCA for 36 h. **(F, G)** Distribution of SK-BR-3 cells, treated for 24 h with 0, 40, 60, and 80 μM of DCA, in the G0/G1, S, and G2/M phases, determined using flow cytometry. **(H, I)** SK-BR-3 cells were stained with Annexin V/PI and analyzed by flow cytometry after being treated with for 24 h with 0, 40, 60, and 80 μM of DCA. The percentage of apoptotic cells is shown in the histogram. The different quadrants Q1, Q2, Q3, and Q4 represent necrotic cells, early apoptotic cells, late apoptotic cells, and viable cells, respectively. Results are presented as means ± SEM from at least three independent experiments. *P < 0.05, **P < 0.01 vs. control in each group. ns, no significance.

To quantify the distinctions corresponding to the breast tumor status and health, we performed the Wilcoxon rank-sum tests with CLR transformation data for discovering BC-related biomarkers in the tissue and gut microbiome, respectively. Among the more notable genera, *Clostridium_XlVa* is enriched in BC patients both in the tissue and gut environment and has the potential to be a biomarker of BC (**Figures 6A, B**).

We attempted to assess the proliferation role of *Clostridium-*specific DCA in the human breast cancer cells MDA-MB-231 and SK-BR-3 by performing the CCK8 assay. DCA increased the proliferation of HER2-positive SK-BR-3 cells compared with that of untreated cells (control), exerting the strongest effect at 80 μM (**Figure 6C**). However, no significant role of DCA was determined in triple-negative MDA-MB-231 breast cancer cell proliferation (**Figure 6D**). As shown in **Figure 6E**, the number of colonies increased in cells treated for 36 h with 80 μM of DCA compared with that in untreated cells. We subsequently verified, through flow cytometry analysis, whether the DCA-induced proliferation of breast cancer cells was associated with variations in the cell cycle. Treatment with DCA (0, 40, 60, 80 μM) decreased the G0/G1 phase percentage from 36.0% to 27.1% and increased the S phase percentage from 31.4% to 43.3% in a dose-dependent manner (**Figures 6F, G**), thus facilitating G1/S progression. Furthermore, no significant effect of DCA was observed in the apoptosis of HER2-positive BC cells. The total apoptotic cell rates of SK-BR-3 cells were 3.3%, 3.0%, 2.5%, and 2.5% (**Figures 6H, I**). These data suggested that DCA stimulates the proliferation and growth of HER2-positive BC cells and promotes cell cycle progression into the S phase but does not contribute to HER2-positive BC cell apoptosis.

### Quantitative proteomic analysis revealed changes in protein expression in DCA-treated SK-BR-3 BC cells

**Figure 7:**
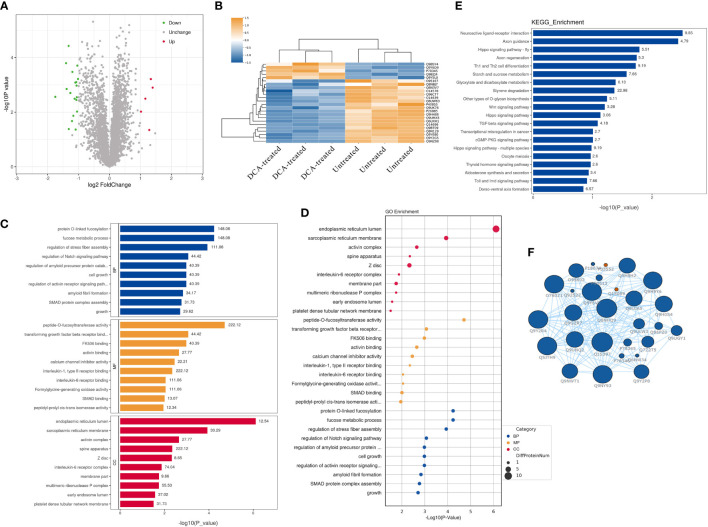
Quantitative proteomic analysis of SK-BR-3 breast cancer cells from DCA-treated and untreated control groups. **(A)** The volcano plot shows the variation in protein expression. Proteins significantly upregulated are labeled red and those significantly downregulated were labeled green. Gray spheres indicate no significance between the group differences, *P* > 0.05. **(B)** Hierarchical clustering of differentially expressed proteins between the DCA-treated and untreated group, *n* = 3 samples for each group (three data points for each bar). Each row represents one significant protein. The color key represents the log2-transformed intensity values of each protein. **(C, D)** The top 10 significantly enriched gene ontology terms in BP (blue), MF (orange), and CC (red). The numbers above the bar charts represent the richFactor. **(E)** The top 20 significantly enriched KEGG pathways and the richFactor shown on bar charts. **(F)** Protein–protein interaction network of the proteins in the module. The blue node indicates the most strongly upregulated protein, and the red node indicates the most strongly downregulated protein. The larger the area of the nodes, the more important the nodes. BP, biological processes; MF, molecular functions; CC, cellular components.

We performed quantitative tandem mass spectrometry experiments using 4D label-free labeling of DCA-treated and untreated SK-BR-3 BC cells. In total, we identified 5,331 proteins in both groups. Of these, 24 differentially expressed proteins (5 upregulated and 19 downregulated) were identified (*P* < 0.05 after *t*-test, fold change ≥2.0, **Figure 7A**). The heatmap of the hierarchical cluster analysis showed that these proteins were well distinguished, which helped clearly visualize the changes in protein expression (**Figure 7B**). GO and KEGG pathway enrichment analyses were performed between DCA-treated and untreated SK-BR-3 BC cells. The most enriched GO terms of biological processes (BP), molecular functions (MF), and cellular components (CC) were annotated as protein O-linked fucosylation (two proteins, richFactor = 148.08), peptide-O-fucosyltransferase activity (two proteins, richFactor = 222.12), and endoplasmic reticulum lumen (seven proteins, richFactor = 12.54), respectively (**Figures 7C, D**, **Table 3**). The results of the KEGG pathway analysis reveal that neuroactive ligand–receptor interaction emerged as the most drastically enriched pathway and was linked to three upregulated proteins in DCA-treated BC cells, namely, nicotinic acetylcholine receptor alpha-9, P2X purinoceptor 5, and partitioning defective protein 3 (**Figure 7E**, **Table 3**). A complex network of proteins with high expression and significant differences in expression is shown in **Figure 7F**.

**Table 3:** Distribution of proteins and signaling pathways after DCA treatment based on GO and KEGG analyses.

Database	Term	Count	*P*-value	FDR	richFactor	Protein names
GO	(BP) protein O-linked fucosylation	2	5.81203E−05	0.0041	148.08	POFUT1 (O-fucosyltransferase 1), POFUT2 (O-fucosyltransferase 2)
	(MF) Peptide-O-fucosyltransferase activity	2	1.94269E−05	0.0028	222.12	POFUT1 (O-fucosyltransferase 1), POFUT2 (O-fucosyltransferase 2)
	(CC) endoplasmic reticulum lumen	7	7.74755E−07	0.0002	12.54	FKBP7 (peptidyl-prolyl cis-trans isomerase FKBP7), ERAP1 (endoplasmic reticulum aminopeptidase 1), SUMF2 (inactive C-alpha-formylglycine-generating enzyme 2), POGLUT2 (O-glucosyltransferase 2), CNPY3 (canopy homolog 3), CNPY4 (canopy homolog 4), FKBP2 (peptidyl-prolyl cis-trans isomerase FKBP2)
KEGG	Neuroactive ligand–receptor interaction	3	0.003067213	0.2688	9.85	CHRNA9 (nicotinic acetylcholine receptor alpha-9), P2RX5 (P2X purinoceptor 5), PARD3 (partitioning defective protein 3)

BP, biological processes; MF, molecular functions; CC, cellular components; FDR, false discovery rate.

## Discussion

Multiple studies worldwide have provided evidence on the close association between BC and microbial dysbiosis (Xuan et al., 2014; Goedert et al., 2015; Hieken et al., 2016; Costantini et al., 2018; Plaza-Diaz et al., 2019; Nejman et al., 2020; Bobin-Dubigeon et al., 2021; Byrd et al., 2021), and the abundances of specialized microorganisms are also correlated with an immune signature and prognostic characteristics (Gopalakrishnan et al., 2018; Terrisse et al., 2021; Tzeng et al., 2021). However, there exists a significant lack of agreement in the types of breast tumor-associated microorganisms identified in published studies. Here, we first presented a large-scale, integrative meta-analysis on public 16S rRNA datasets to characterize the breast tissue and gut microbiota signatures of patients with breast tumors and assess the potential of important microbial signatures for distinguishing BC from health statuses. Compared with datasets from a single study, pooled datasets of breast tissues and gut microbiota from multiple studies enabled the identification of microbiota signatures more accurately and comprehensively.

This meta-analysis revealed significant changes in the microbial flora of patients with BC, and we also found that critical microbial characteristics can be used to diagnose BC. In pooled RF models, specific microbes from malignant breast tissue could be used to discriminate between BC and non-cancer populations and yielded AUCs of 74.27% in the training module. Cohort heterogeneity is critical in trans-cohort generalization. In our work, important microbial features were validated for effectiveness *via* cohort-to-cohort transfer (average AUC = 0.65) as well as LOCO validation (average AUC = 0.66) while underscoring the adverse impact of technical and geographical discrepancies on the model’s generalizability. Consistent with our hypothesis, microbial components are similar in malignant breast and lesion-adjacent tissues. The AUC was 75.29% in cancer-adjacent tissues vs. non-cancer control RF models, which was similar to that in cancer vs. non-cancer population models (AUC = 74.27%). The result indicates that the lesion-adjacent tissue microbiome is sufficient for BC prediction, and lesion-adjacent tissues could be used for sampling during BC screening.

It is generally known that intestinal microbes actively participate in the development of various cancers, such as ovarian (Cheng et al., 2020; Laniewski et al., 2020), prostate (Fujita et al., 2022; O'Rourke, 2022), breast (Ingman, 2019; Hossain et al., 2021; Zhang et al., 2021), and colorectal cancers (Wong and Yu, 2019; Song et al., 2020; Rebersek, 2021). The known microbial mechanisms of action can modulate the tumor microenvironment by regulating the non-hematopoietic and hematopoietic components of the intestinal epithelial barrier and primary and secondary lymphoid organ activities, thereby affecting cancer occurrence and development (Sepich-Poore et al., 2021). Recent findings have revealed that mammary microbiota may be derived from the gut. Intestinal bacteria have easy access to the mammary gland *via* the entero-mammary pathway. This mechanism involves CD18^+^ cells and dendritic cells, which have the capacity to transmit intestinal microbes into the mammary gland (Angelopoulou et al., 2018; Chadha et al., 2021; Wang et al., 2021). In the present study, we showed that the abundance of *Bacteroides* in patients with BC was significantly higher than that in controls. This result is consistent with the findings from previous studies, which suggested *Bacteroides* associations with BC (Zhu et al., 2018; Byrd et al., 2021; Hou et al., 2021). Enterotoxigenic *Bacteroides fragilis* accelerated tumor cell growth in the mammary glands or intestines and metastatic progression in breast ducts using secretory *B. fragilis* toxins. Parida et al. (2021) showed that compared with control mice, mice harboring gut enterotoxigenic *B. fragilis* showed an almost 3.3-fold increase in the tumor volume over 7 weeks. It is promising that not all bacteria species are detrimental. *Prevotella* is considered a beneficial bacteria based on its low levels in patients with BC and is the only co-differential bacterium present in breast tissues and the intestinal environment in cancer and non-cancer populations. Evidence from existing literature suggests that *Prevotella* potentially exerts an antitumor effect through its metabolites. Acetic acid, the major metabolic product of *Prevotella*, significantly inhibited the synthesis of nitric oxide in AGS cells (Dong et al., 2017), which could enhance protooncogene expression and inhibit the apoptosis of tumor cells (Li et al., 1997; Ambs et al., 1998).

With respect to cancer diagnosis, critical intestinal microbial characteristics can help discriminate between cancer and non-cancer populations. In our study, RF models constructed using data on important gut microbiota (AUC = 68.08%) could provide insights on BC diagnosis. However, gut microbiota-related RF models yield a lower accuracy than tissue microbes in distinguishing cancer from non-cancer controls (AUC = 74.27%). This is likely because tissue-based samples are invasive and assess microbial structure at the lesion site more accurately than stool-based samples. Zhu et al. (2018) showed that the training set of fecal samples performed remarkably when patients with BC and healthy controls were compared, and the AUC value achieved was 85.52%, which was greater than the value obtained in the present study. One explanation is that in the stool samples from multiple studies, ethnic heterogeneity, varied dietary habits, and different exposure factors within groups could contribute to the discrepancies in the intestinal microbial communities, thus affecting model performance. Furthermore, the performance is potentially influenced by the different sequencing procedures, such as shotgun metagenomic sequencing and 16S rRNA sequencing. Shotgun metagenomic sequencing data are well-recognized to be advantageous for the identification of special microbes up to the species and even the strain level, which is more conducive to improving accuracy in BC diagnostic models. More importantly, the AUC value of the BC health model based on stool microbiota reported by Zhu et al. was greater than 80%, which further illustrates that the specific intestinal microbiota has BC diagnostic potential comparable to breast tissue microbiota, even if it is present far from the breast lesion site.

Currently, BC diagnosis is mainly based on imaging examinations, and mammography, magnetic resonance imaging (MRI), ultrasound imaging, and computed tomography (CT) are the most common methods used in clinical applications, with a sensitivity of more than 80% (Jafari et al., 2018). However, despite the various benefits of imaging techniques, they have some limitations, such as costs and potential radiation risks. The most important caveat is that imaging modalities may yield false-positive and false-negative errors. For example, if lesions labeled as BI-RADS category ≥4b are considered to be malignant, the rates of misdiagnosis under mammography, ultrasound, and MRI would be 17.9%, 17.5%, and 35.3%, respectively (Tan et al., 2019). Although the AUCs are low owing to the influence of the sequencing platform, region, and other factors, BC and non-cancer states can be assessed using characteristic microorganisms. Particularly, gut microbes, as a non-intrusive and convenient method, are easily applicable in clinical settings.

As for the BC-associated biomarker *Clostridium_XlVa*, Huang et al. (2020) provided that *Clostridium XIVa* was enriched in non-small hepatocellular carcinoma. Based on the transcriptome and fecal microbiota data, the increased abundance of *Clostridium XIVa* accompanied by a decrease in CCL21, which facilitates tumor growth, indicates an unfavorable prognosis. *Clostridium XIVa* is highly potent in enhancing Treg cell abundance and inducing important anti-inflammatory molecules such as interleukin-10 (Atarashi et al., 2013), as well as affecting bile acid-controlled NKT cell accumulation. Changes in the *Clostridium XIVa* may result in alterations of the tumor microenvironment, which highlight the potential of *Clostridium XIVa* as a biomarker for breast cancer. Recent data suggest that the human microbiota contributes to several common types of cancer, not only by playing procarcinogenic roles of specific pathogens but also *via* the influence of their metabolome. For example, the intestinal microbiome–metabolite formate drives CRC tumor invasion *via* triggering AhR signaling while increasing cancer stemness (Ternes et al., 2022). Obesity induces changes in the gut microbiota, thereby increasing the levels of the gut bacterial metabolite DCA. The enterohepatic circulation of DCA stimulates the SASP phenotype in hepatic stellate cells, which in turn secretes various inflammatory and protumor factors in the liver (Yoshimoto et al., 2013). (Costarelli and Sanders, 2002) reported that plasma DCA concentration in patients with breast cancer was 52% higher than that in healthy women, supporting the hypothesis that DCA might be related to the occurrence and development of breast cancer. In this study, we demonstrated that *Clostridium-*specific DCA plays a molecule type-specific role in the proliferation of BC cells, significantly promoting the proliferation of HER2-positive BC cells but not affecting triple-negative BC cells. As a secondary metabolite of bile acids, DCA increases ROS production and DNA damage, which could cause K-RAS mutations, aneuploidy, and micronuclei formation (Si et al., 2021). Bile acids are also present in breast tissues and may originate from the gut (Rezen et al., 2022). Findings from proteomics analysis indicate that DCA promotes the activation of peptide-O-fucosyltransferase activity and triggers the neuroactive ligand–receptor interaction pathway, which may play a role in cancer progression. Meanwhile, *POFUT1* and *POFUT2* genes, involved in cancer-related pathways, have been screened. The overexpression of *POFUT1* leads to Notch1 signaling dysregulation associated with poor prognosis (Wan et al., 2017; Deschuyter et al., 2020). Leng et al. (2018) stated that genes encoding proteins associated with fucosylation, including *POFUT1* and *POFUT2*, may serve as circulating biomarkers of lung cancer. Objectively, the findings suggest that *POFUT1* and *POFUT2* are worthy of further investigation for their functions in BC.

Unlike previous studies, ours is the first to use comprehensive datasets to investigate the microbial community in both breast and gut samples of patients with BC. Based on the findings from the meta-analysis of eight studies, the interference of biotechnology and regional differences were reduced, and we identified a consistent and reliable variation in the microbiome of patients with BC. Additionally, important microbial features of the tissue or intestinal environment were useful for the diagnosis of BC and could provide a new approach for cancer screening. We recognize that the study had limitations in validation, such as the lack of additional validation using actual clinical patient samples. To overcome this drawback, we strived to strengthen the evidence from other perspectives of the study design and used interstudy cross-validation and within-study validation simultaneously to verify the stability of important microbial features in BC diagnosis. Lastly, the interaction between microorganisms and the occurrence and progression of cancer is very complex, and a variety of bacteria or its metabolites may promote or inhibit the development of breast cancer. Herein, we only examined the effect of *Clostridium*-specific metabolite DCA on the proliferation, cycle, and apoptosis of breast cancer cells. More microorganisms and its metabolites are still worth exploring. In our future studies, external validation in large clinical populations will be necessary, and we would also like to integrate additional factors, such as lymph node metastasis, Ki67 expression, molecular subtypes of breast cancer, and progression-free survival to determine the microbiome associated with breast cancer prognosis.

By re-analyzing raw 16S rRNA gene sequence data from fecal and tissue samples, we suggested a strong association between the microbiota and breast tumorigenesis. The overlapping specific microbes of the breast tissue and gut environment may provide biomarkers of BC, and the similarities between the carcinoma and adjacent normal tissues indicate that microorganisms might pave the way for our understanding of microbial roles in carcinogenesis. We also found that the *Clostridium*-specific metabolite DCA could stimulate proliferation by promoting cell cycle progression into the S phase. From the perspective of disease diagnostics, important microbial features could aid BC screening at early stages. In conclusion, the findings of this study provide a novel perspective for further insights on the role of the microbiome in BC tumorigenesis and progression.

## Data availability statement

The datasets presented in this study can be found in online repositories. The names of the repository/repositories and accession number(s) can be found in the article/supplementary material.

## Author contributions

JX and TS conceived and designed the article. NW and JX wrote the draft of the paper. WH and MH collected and organized the published 16S rRNA sequencing data. NW and XL analyzed the metagenomics data. NW and JY completed the *in-vitro* cell experiment. LJ, HC, MJ, TS, and JX participated in the manuscript revision. All authors contributed to the article and approved the submitted version.

## Supplementary material

Supplementary Figure 1-2 The total variance explained by disease status (BC, benign tumor, health) are plotted against total explained by patient age, BMI in studies using breast tissue and fecal samples.

Supplementary Figure 3-5 Results of the for differential microbial features in lesion-adjacent tissue vs. normal tissue, cancer tissue vs. benign tissue, cancer stool vs. benign stool groups (P<0.05).

Supplementary Table 1-2 Key components of the random forest model constructed in this study.
