# Supplementary material for: Identifying distinctive tissue and fecal microbial signatures and the tumor-promoting effects of deoxycholic acid on breast cancer
Source: Front Cell Infect Microbiol. 2022 Dec 13;12:1029905. doi: 10.3389/fcimb.2022.1029905 (PMC9793878; doi:10.3389/fcimb.2022.1029905)
Supplement: Supplementary Figure 1-2 — The total variance explained by disease status (BC, benign tumor, health) are plotted against total explained by patient age, BMI in studies using breast tissue and fecal samples. [file DataSheet_1.docx]

Supplementary Material

# Supplementary Figures and Tables

## Supplementary Figures


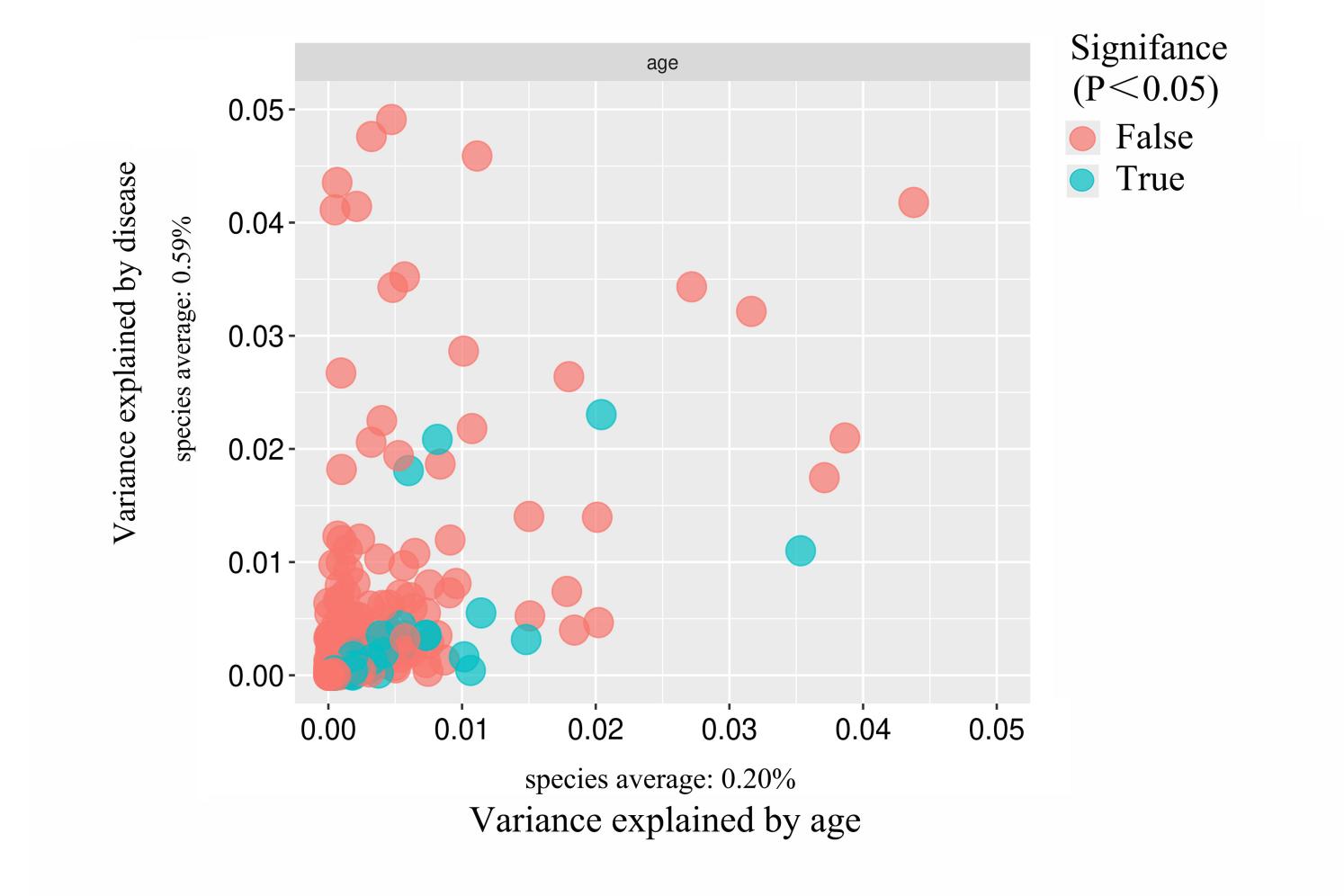


**Supplementary Figure 1.**

The total variance explained by disease status (BC, benign tumor, health ) are plotted against total explained by patient age in studies using breast tissue samples. The significantly differential ASVs are colored in blue and P values were from two-way ANOVA test.


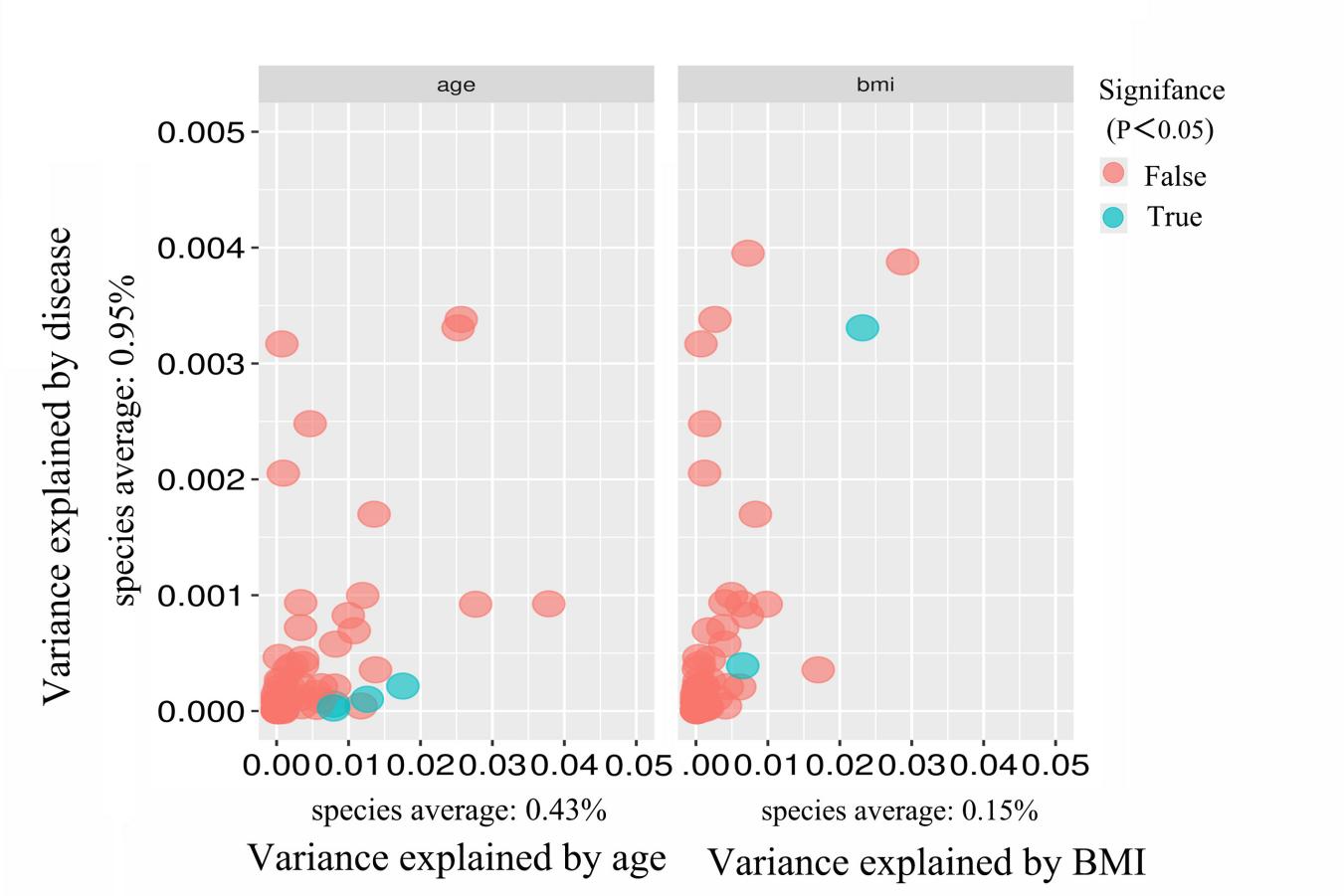


**Supplementary Figure 2.**

The total variance explained by disease status (BC, benign tumor, health ) are plotted against total explained by patient age and BMI in studies using fecal samples. The significantly differential ASVs are colored in blue and P values were from two-way ANOVA test.


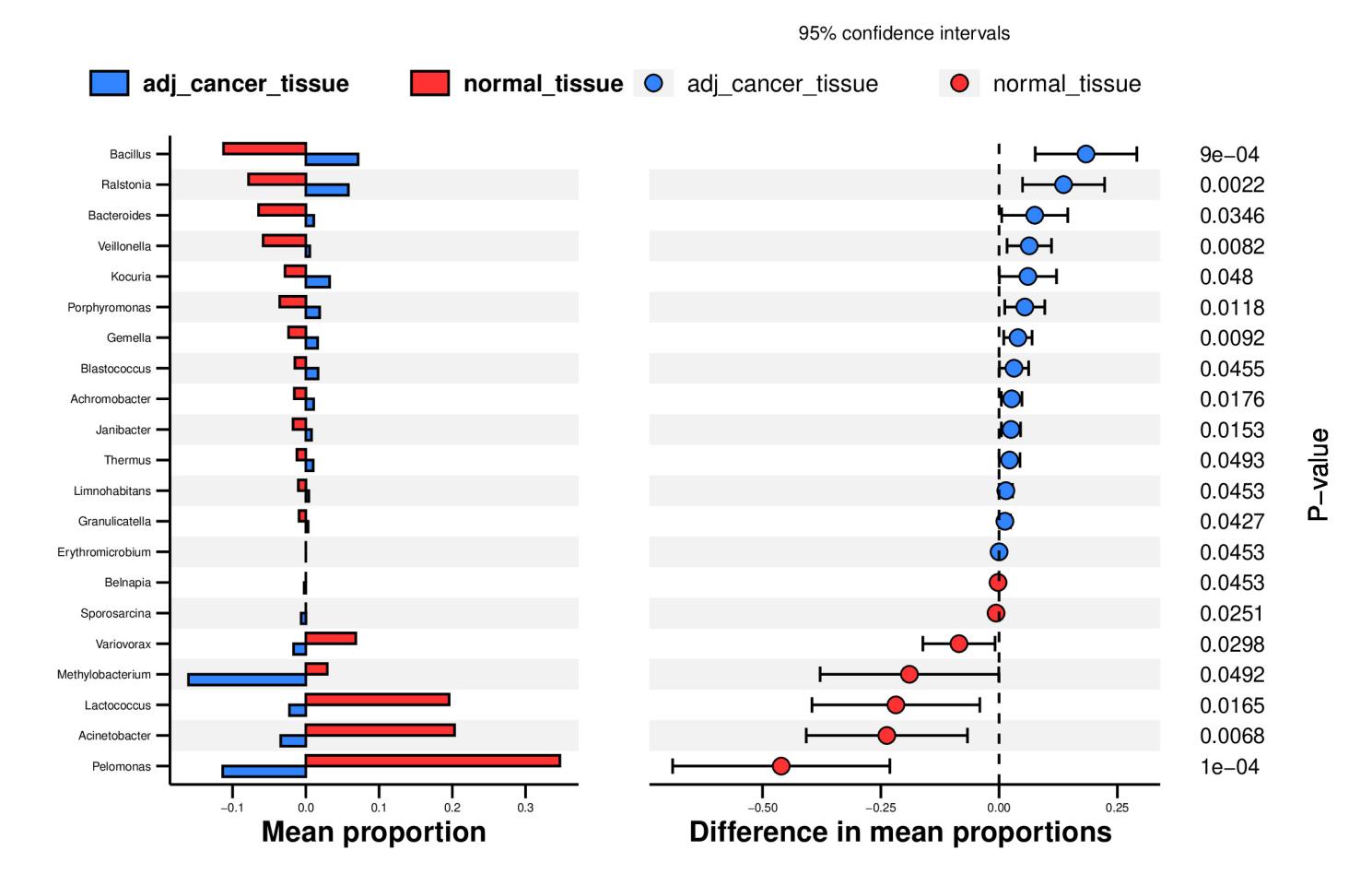


**Supplementary Figure 3.**

The bacteria significant differences between normal and adjacent-cancerous tissue in the breast with 0.95 confidence interval. 14 enriched and 7 depleted genera could be found in adjacent-cancerous tissue.


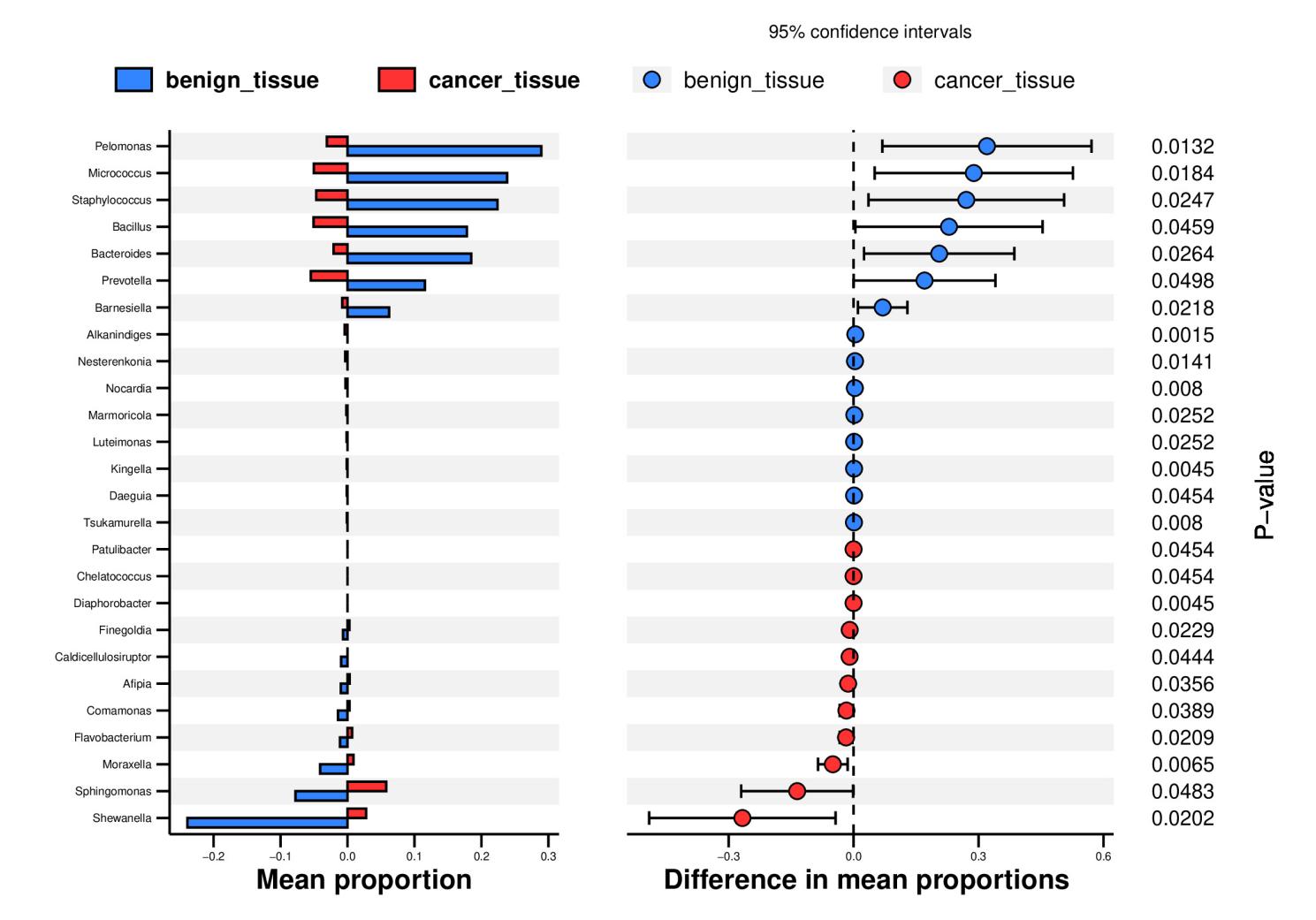


**Supplementary Figure 4.**

The bacteria significant differences between cancerous and benign tumor tissue with 0.95 confidence interval. Eleven enriched and fifteen depleted genera could be found in cancerous tissue


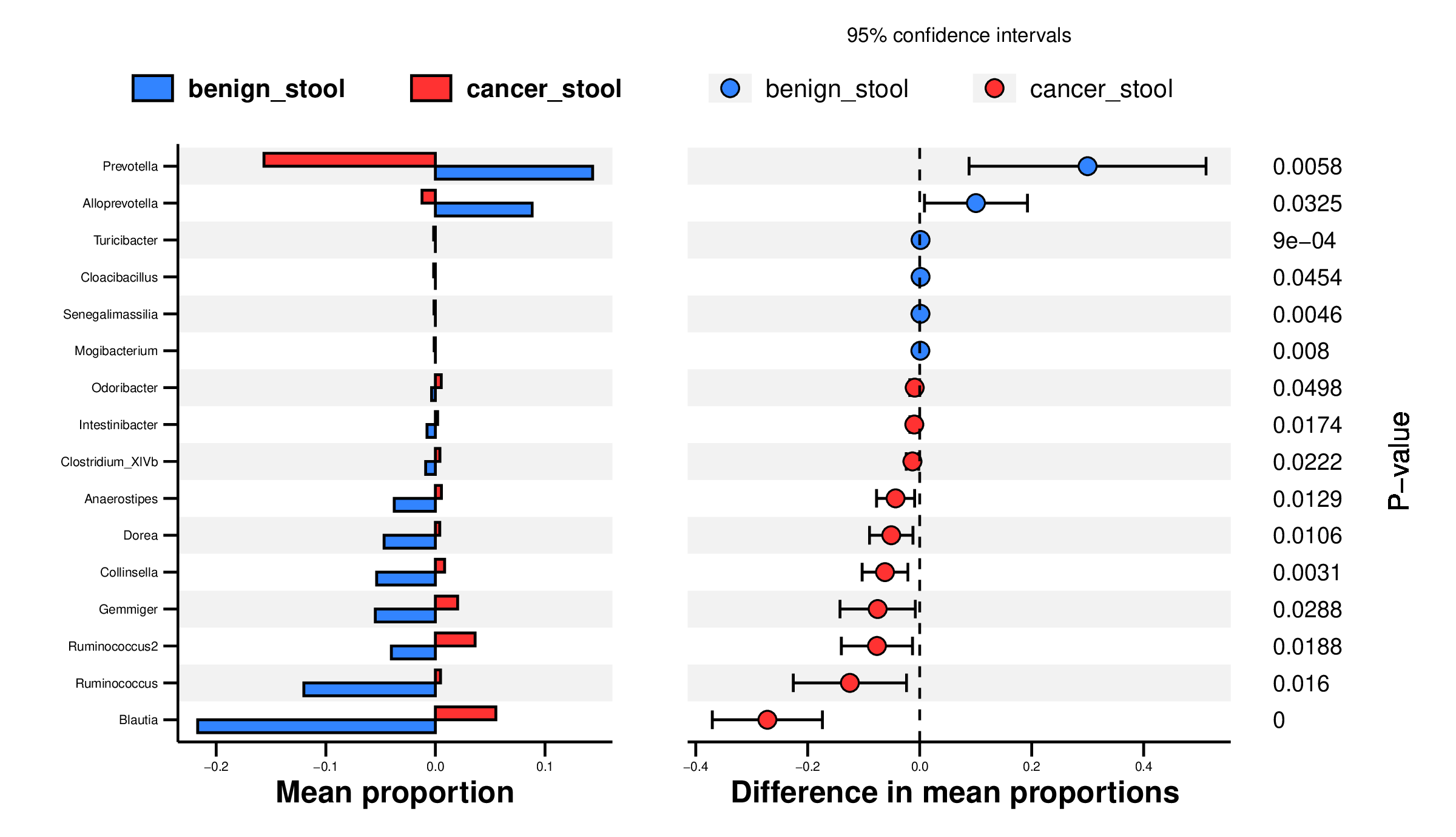


**Supplementary Figure 5**

The bacteria significant differences between breast cancer patients and breast benign tumorpatients with 0.95 confidence interval. Ten enriched and six depleted genera could be found in stool of breast cancer group.

Table S1 : Important features in random forest model of cancer_tissue vs non-cancer_tissue

| Feature_id | MeanDecreaseAccuracy |
| --- | --- |
| Prevotella | 7.92256566 |
| Reyranella | 7.414796597 |
| Atopobium | 6.878778418 |
| Micrococcus | 6.792889592 |
| Butyrivibrio | 6.751985578 |
| Weissella | 6.403706115 |
| Ruminococcus | 6.196978037 |
| Hydrotalea | 6.018145224 |
| Paenibacillus | 5.873667295 |
| Yersinia | 5.599143285 |
| Geobacillus | 5.53275211 |
| Aeribacillus | 4.951977946 |
| Rahnella | 4.560116684 |
| Sphingobacterium | 4.52013516 |
| Ralstonia | 4.27426712 |
| Escherichia.Shigella | 4.067390145 |
| Thermoanaerobacterium | 3.881685185 |
| Lachnospiracea_incertae_sedis | 3.496327547 |
| Blastococcus | 3.198852416 |
| Fusicatenibacter | 3.166369999 |
| Caldanaerobius | 3.14955733 |
| Leptotrichia | 2.963029452 |
| Lactobacillus | 2.877443597 |
| Desulfitobacterium | 2.836869053 |
| Rothia | 2.830308328 |
| Roseburia | 2.741324613 |
| Pseudobutyrivibrio | 2.685776864 |
| Bacteroides | 2.671658678 |
| Alistipes | 2.639718894 |
| Sphingomonas | 2.615058739 |

Table S2: Important features in random forest model of cancer_stool vs non-cancer_stool

| Feature_id | MeanDecreaseAccuracy |
| --- | --- |
| Bacteroides | 19.42141373 |
| Prevotella | 11.35314251 |
| Haemophilus | 7.530259743 |
| Butyrivibrio | 6.612070197 |
| Fusobacterium | 6.521330882 |
| Faecalibacterium | 5.562758217 |
| Ruminococcus | 5.406244888 |
| Phascolarctobacterium | 5.378387885 |
| Barnesiella | 5.270628584 |
| Alloprevotella | 5.246599758 |
| Intestinimonas | 4.960054046 |
| Blautia | 4.251599965 |
| Parabacteroides | 4.136353663 |
| Anaerostipes | 3.992872805 |
| Clostridium_XlVa | 3.849195589 |
| Clostridium_IV | 3.753102135 |
| Succinivibrio | 3.702811869 |
| Howardella | 3.662358112 |
| Dialister | 3.549975122 |
| Clostridium_XVIII | 3.434105505 |
| Lactococcus | 3.316607884 |
| Parasutterella | 3.193339149 |
| Gemmiger | 3.191948567 |
| Coprococcus | 3.145439883 |
| Lachnospiracea_incertae_sedis | 2.949925027 |
| Pantoea | 2.88388325 |
| Campylobacter | 2.701916451 |
| Roseburia | 2.588855999 |
| Slackia | 2.579226417 |
| Faecalitalea | 2.501719901 |
